# Millennial Floristic Diversity and Land Management as Inferred from Archaeo-Palynological Research in Southern Italy

**DOI:** 10.3390/plants14091367

**Published:** 2025-04-30

**Authors:** Eleonora Clò, Anna Maria Mercuri, Jessica Zappa, Cristina Ricucci, Lorenzo Braga, Assunta Florenzano

**Affiliations:** Laboratorio di Palinologia e Paleobotanica, Dipartimento Scienze della Vita, Università degli Studi di Modena e Reggio Emilia, 41125 Modena, Italy; eleonora.clo@unimore.it (E.C.); annamaria.mercuri@unimore.it (A.M.M.); cristina.ricucci@unimore.it (C.R.); lorenzo.braga@unimore.it (L.B.); assunta.florenzano@unimore.it (A.F.)

**Keywords:** pollen, botany, biodiversity, database, cultural landscapes, land-use, human impact, palaeoecology, sustainability

## Abstract

Palynology is an invaluable tool for reconstructing past biodiversity in agrarian and cultural landscapes and for understanding present-day environmental assets. By analysing past evidence, rooted in botanical knowledge, we can foresee future environmental trends. Italy, at the centre of the Mediterranean, is one of the richest countries in terms of pollen analyses from archaeological sites and therefore is particularly suited to reconstructing human–environment relationships and anthropogenic impacts on flora over time. We selected data filled in the database BRAIN. This paper presents new elaboration on pollen data from 14 published and unpublished archaeological sites, showing past plant diversity and land management in prehistorical and historical contexts of southern Italy. Overall, the research demonstrates that the floristic palaeodiversity, as revealed through the group-equalised indicator species analysis, supports and validates the palynological data on the flora of Campania, Basilicata, and Sicily. The study highlights the presence of ubiquitous pollen taxa in anthropogenic environments and explores the connection between past and present plant diversity.

## 1. Introduction

A comprehensive understanding of plant diversity is essential for advancing research in systematics, chorology, phytogeography, and ecology [1,2,3,4]. Floristic inventories, which document the plant species present in a given region, offer essential information that supports effective decision-making in biodiversity conservation and landscape management [5,6,7]. These inventories not only provide insights into species distribution and ecosystem structure but also serve as key references for assessing environmental changes, identifying conservation priorities, and guiding sustainable land-use planning strategies. As such, floristic surveys are essential tools for both the scientific community and policymakers, facilitating the protection and management of plant diversity and the ecosystems that rely on it.

While much of our current understanding of plant diversity patterns derives from studies of local, regional, and national floras, as well as species distribution maps [8,9,10], the palynological approach provides a valuable complementary perspective [11,12,13]. By analysing pollen records, the reconstruction of past vegetation dynamics becomes possible, also offering a more detailed understanding of plant diversity over time.

Palaeoecological studies often examine off-site records such as marine, lake, or peat cores, as well as trenches [14,15,16,17,18], while historical research typically focuses on on-site contexts, like archaeological layers [19,20]. Both approaches help to fully understand the current dynamics of biodiversity and ecosystems, and the long-term, human-induced changes in natural resource exploitation that have shaped the environment over millennia [21]. This is particularly relevant in the Mediterranean Basin, which is the second largest biodiversity hotspot in the world [22]. The region’s unique species assemblages and diverse ecological niches have made it a focal point for both human and biological evolution throughout history [23,24,25].

Over millennia, the unique biodiversity and varied ecological niches of the Mediterranean have shaped patterns of human distribution, agricultural practices, and the sustainable (or unsustainable) exploitation of natural resources [26,27,28,29]. The understanding of Long-Term Environmental Changes (LoTEC) implies knowledge and description of environmental conditions at different stages of human impact, providing insights into the scale and duration of human presence on a territory [28,30]. LoTEC can be analysed by examining human choices on tree crops and synanthropic plants, which thrive in rural and urban settings [31,32]. Complementarily, the “AP/NAP” ratio, which compares the percentages of arboreal (trees, shrubs, and lianas) and non-arboreal (herbs) pollen, is used to reconstruct trends in forest cover, including human-induced deforestation [33,34]. Fire history completes the reconstruction of environmental transformations as natural dynamics or land-use management (e.g., [35,36,37,38]).

Notably, while human populations have played a significant role, the evolution of Mediterranean landscapes has also been profoundly shaped by climate variability [39,40,41,42,43,44,45,46]. The interaction between adaptive responses to climate change and local environmental and socio-cultural variables has been a defining factor in the transformation of the Mediterranean area, especially throughout the Holocene [47,48,49,50]. These dynamic processes have continuously modified the region’s ecosystems, influencing plant species distribution, altering habitat types, and driving the distribution of its biodiversity. In this context, pollen morphology offers a valuable means of gaining detailed insights into plant biodiversity, with palynological research focusing on biodiversity trends as a primary goal [51,52,53].

### Aim of the Paper

To illustrate past biodiversity as revealed by palynological analyses of past contexts, we selected pollen studies carried out on archaeological sites distributed across southern Italy. By employing a biological lens, our research seeks to enhance the understanding of floristic diversity that has historically characterised this area and has been impacted by the ongoing human pressure over an extended period (millennial scale). Actually, palynology is a multi-levelled science, and several scientific layers can be envisaged in the research on one-single site. Palynological literature stated how distinguishing the ecological implications of different pollen types from palaeoenvironmental assemblages (e.g., [19,54]). The less explored field, however, is the floristic level of pollen analysis, due to the objective difficulties that pollen identification may encounter (e.g., [55,56]; pollen atlases such as: [57,58,59]).

The aim of this paper is to explore the floristic level of palynology of archaeological sites, and to report the most detailed floristic inventory obtained from archaeo-palynology to date. To achieve this goal, two key prerequisites were necessary: (a) full control over pollen identification and consistent application of palynological classification criteria across all sites; (b) holistic interpretative approach grounded in botanical expertise, encompassing both pollen taxonomy and ecological understanding. Therefore, this research consists of the original data elaboration of pollen analyses carried out by the authors. Pollen data from published sites from Campania, Basilicata, and Sicily were selected, and unpublished data from two sites in Sicily were also added.

Through a long-term ecological perspective, data shed light on the effects of human activity and climate changes on the natural environment, including shifts in species presence and distribution. Additionally, the paper offers insights into the resilience and adaptation of plant species in response to such pressures, contributing to a more comprehensive picture of southern Italy’s flora in both past and contemporary contexts.

## 2. Results

### 2.1. Overview of Sites in Southern Italy

**Figure 1:**
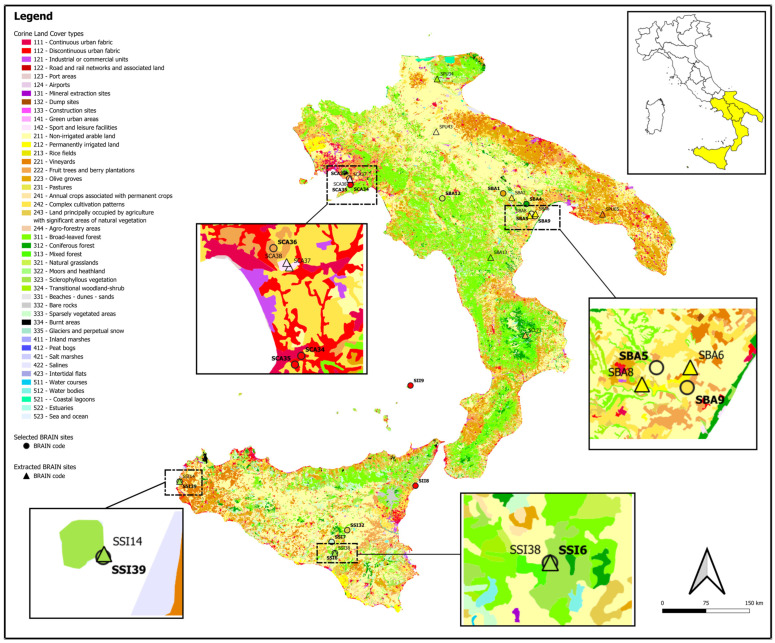
Map showing the 26 sites extracted from the BRAIN database, each associated with its unique BRAIN ID. The sites selected for this research are represented by points on the map with their ID evidenced in bold, while those that were queried (extracted BRAIN sites) but not included in these data elaborations are shown as triangles. The colour of each site matches with the Corine land cover classification [83].

The results obtained from querying the BRAIN database show 26 sites with pollen analyses in five regions (https://brainplants.successoterra.net/; [60] accessed on 25 February 2025). The most represented region is Basilicata, with 9 sites [61,62,63,64,65,66,67,68,69], followed by Sicily (8 sites; [70,71,72,73,74,75]), Campania (5 sites; [76,77]), Apulia (3 sites; [78,79,80]), and Calabria [81,82], which has one site (Figure 1).

Most of the sites are included in the “on-site” category. There is only one off-site in Basilicata (a terrestrial core; SBA13—Stagno del Pesce, STAPE) and two spot records in Campania, from Pompeii (two plaster samples from the houses “Casa del Menandro” and “Casa del Centenario”; SCA37 and SCA38, respectively). The on-sites are mostly settlements, both rural and urban, but there are also monumental buildings and sacred/funerary complexes. The sites date back from the Mesolithic to the Middle Ages chrono-cultural phases, with most of the contexts attributable to the Roman Age. For details, refer to Table S1.

### 2.2. Case Studies Selected from Different Regions of Southern Italy

Below, according to the criteria mentioned above (Section Aim of the Paper), 14 sites have been selected as suitable for data elaboration thanks to the high number of samples and well-studied contexts. They are all archaeological sites, and are listed, from north to south, according to their region—Campania, Basilicata, and Sicily—and BRAIN ID entry number. For each site, the main chronology and a summary of the palynological research results are described. Table 1 reports an overview of the main results from these case studies. The metadata and main palynological references of the sites selected for this study are presented in Table S1.

#### 2.2.1. Campania

*SCA34—Stabiae—Villa San Marco*—Stabiae, located between the Vesuvian area and the Sorrento Peninsula, flourished from its destruction by Sulla (89 BC) until the eruption of Vesuvius (AD 79). During this period, many luxurious villas with beautifully decorated apartments, thermal baths, and porticoes, including Villa San Marco and Villa Arianna (see next paragraph), were built. At Villa San Marco, pollen samples were collected from soil buried by the AD 79 lapilli and ash deposits in the East Portico Garden. Pollen spectra show a significant plant diversity, even higher than at Villa Arianna [77]. This reflects vegetation that included deciduous broadleaf forests and Mediterranean macchia with evergreen sclerophylls and patches of pine forests. Asteraceae and Fabaceae, both including several pollen taxa, could have been associated with gardens or pastoral/livestock farming activities. However, human activities in the territory are mainly represented by fruit trees (*Juglans regia*, along with *Olea europaea* and *Castanea sativa*) and ornamental trees (e.g., *Platanus orientalis*).

*SCA35—Stabiae—Villa Arianna*—Pollen data from soil samples covered by the AD 79 lapilli in the Peristyle H Garden reveal a mix of natural flora and ornamental or utilitarian plants, suggesting an anthropogenic landscape characterised by arboriculture [76,77]. The high presence of cereal pollen may indicate that plant processing has occurred locally, while species from the cabbage and daisy families could have been cultivated as ornamentals and food. Additionally, the presence of *Matthiola* pollen raises the possibility that the plant was either grown in the garden or transported from the coastal area of the Gulf of Naples.

*SCA36—Pompeii—Civita Giuliana*—Civita Giuliana is situated in the outskirts of Pompeii, an area with several Roman villas used for both productive and residential purposes. In this context, pollen samples were collected from farmland with evidence of planting beneath the AD 79 lapilli layer. Pollen spectra are composed of both natural and synanthropic taxa, reflecting an open landscape influenced by human activities, such as the cultivation or the processing of wheat and barley (*Avena/Triticum* and *Hordeum* groups). Additionally, Brassicaceae were likely cultivated as vegetable plants. The evidence also indicates that cultivated plants included ornamental species (e.g., *P. orientalis* and *Myrtus communis*) and fruit trees (e.g., *O. europaea*, *Corylus avellana*, *Prunus*, and *J. regia*). Evergreen Mediterranean trees and shrubs were also grown at Civita Giuliana [77].

#### 2.2.2. Basilicata

*SBA1—Altojanni*—The site includes an extended rural area between the Bradano river and Bilioso torrent, mainly occupied during the Roman and Medieval periods. Samples were retrieved from both archaeological layers and built structures within the site. Altojanni was characterised by an open landscape. Mixed broadleaf forests with oaks, *C. avellana*, *Fraxinus*, *Ulmus*, and *Fagus sylvatica* lived on cooler slopes. During the Medieval period, wet environments with freshwater plants expanded, suggesting hydrological changes or water management practices. The cultural landscape was shaped by agriculture (*Avena/Triticum* group, *Hordeum* group, and *Secale cereale*) and grazing (Cichorieae and coprophilous fungal spores related to high density of herbivores). Grazing and browsing promoted the expansion of Chenopodiaceae/Amaranthaceae, *Centaurea nigra* type, Poaceae wild grass group, *Trifolium* type, Fabaceae, and Apiaceae. Other human-related indicators included *Plantago* (trampling areas) and *Urtica dioica* (organic-rich soils). In general, palynological evidence highlights a landscape influenced by land-use changes from Roman agriculture to Medieval intensive grazing [61,62,68].

*SBA4—Difesa S. Biagio*—It is located on a calcareous hill overlooking the Metapontine plain and one of the biggest rural settlements in the Bradano Valley, occupied from the Middle Bronze Age to the late Hellenistic period. The site, settled long ago by the Enotrians, shows evidence of domestic and agricultural structures, including houses, food storage areas, and an oil press. Pollen samples have been collected from the site area, specifically from a short Eastern Trench located about 70 m from the area of the house structures. The surrounding landscape was predominantly characterised by grassland, with Poaceae, Asteraceae, Chenopodiaceae/Amaranthaceae, and Brassicaceae, indicating a dry-tolerant environment with scarce wetland plants. A xerophilous woodland with *Quercus ilex*, *O. europaea*, and *Pinus halepensis* was quite far from the site. Deciduous *Quercus* and *Carpinus betulus* formed mixed forests on shady slopes, with *Ulmus* and *Alnus* occurring in riparian areas. The cultural landscape was shaped by pastoral activities, as evidenced by Cichorieae and coprophilous fungal spores. By the late Hellenistic period, olive trees and cereals (*Avena/Triticum* group) were cultivated, but the low pollen percentages suggest that orchards and fields were not immediately adjacent to the settlements. Archaeological evidence confirms cereal processing and oil production in the site [62,68].

*SBA5—Fattoria Fabrizio*—This farmhouse, in the *Chora* (rural countryside) of the Greek colony of *Metapontum*, was occupied between the 6^th^ and 4^th^ centuries BC, with evidence of a mixed agro-pastoral economy. Samples were collected from within the perimeter of the farm building, specifically from Rooms 1 and 2. Palynological analyses show that the surrounding landscape mainly consisted of open shrublands, grasslands, and scarce wet environments. Woodland consisted mainly of Mediterranean macchia, with evergreen shrubs, holly oak, and olive trees, though it was less extensive than today. Mixed oakwood was more widespread and likely grew on the wetter slopes of the valley. Cereals were cultivated (*Avena/Triticum* and *Hordeum* groups), and the presence of cereal pollen and other palynomorphs related to grains accumulation in a room of the farmhouse indicate possible grain storage. However, livestock grazing, mainly ovicaprids, played a dominant role in shaping the surrounding landscape as indicated by high levels of pastoral pollen indicators and coprophilous fungi. The presence of *O. europaea*, *C. avellana*, *C. sativa*, and *Prunus* pollen suggests the existence of nearby orchards [62,64,65,68].

*SBA9—Pantanello (Pizzica Pantanello)*—The site is a gravel terrace on a hillside near the Basento and Bradano rivers, and the ancient Greek–Roman urban centre of *Metapontum*, settled in 640 BC. Pollen samples were taken from two trenches dug between the archaeological site at the foot of the terrace and the alluvial infilling of the Basento River valley. The multidisciplinary investigation highlights complex interactions between fluvial, colluvial, and environmental human-influenced processes from the Archaic to the Roman periods. Before the 6^th^ century BC, wetlands dominated the Pantanello area. After the 6^th^ century BC, there was an increase in Chenopodiaceae/Amaranthaceae (salt-tolerant), indicating a gradual reduction in wetlands. Human influence intensified, with cereal fields, vineyards, and olive groves, with scattered broadleaved oakwoods (*Quercus*) and Mediterranean macchia (*Pistacia*, *M. communis*, and *O. europaea*). Moreover, Cichorieae, *C. nigra* type, and Asteraceae pollen increased, suggesting an intensification of pastoral activities. Plant macroremains recovered from a sacred spring of the rural sanctuary at the site confirmed the cultivation of figs (*Ficus*), olives (*O. europaea*), grapevines (*Vitis vinifera*), cereals (emmer wheat, durum/bread wheat, and hulled barley), and legumes (e.g., *Vicia* and *Cicer*) [84]. In the Hellenistic and Roman periods (after the 4^th^ century BC), hygro-hydrophytes (e.g., *Ceratophyllum demersum* and *Sparganium emersum* type) declined and broadleaved oak slightly recovered. By the 2^nd^ century BC, wetlands temporarily expanded due to the site’s abandonment, but cereal, olive, and grapevine cultivation spread by the 1^st^ century BC, also suggesting the spread of agricultural activities in the Metapontine plain [62,66,67,68,69].

*SBA12—Torre di Satriano*—The site today hosts the remains of the Medieval *Satrianum* on a hilltop. Different excavation areas in the plateau at the foot of the hill brought to light Archaic residential buildings, including the “*capanna ad abside*” (apsidal hut), the “*Anaktoron*” (the lord’s palace), and a productive complex; palynological results from these contexts provide insights into the vegetation and land-use changes. In the 7^th^ century BC, grasslands dominated, with Poaceae, Asteraceae, Cichorieae, scattered woodlands of deciduous *Quercus*, *C. betulus*, *Ostrya carpinifolia/Carpinus orientalis* type, *Fraxinus*, and Mediterranean vegetation (*Q. ilex*, *Juniperus*, *Phillyrea*, *Pistacia*, and *Erica*). Wetlands, with Cyperaceae, *Sparganium emersum* type, were less extended. Agriculture (*Avena/Triticum* and *Hordeum* groups) and arboriculture (*O. europaea*, *V. vinifera*, *J. regia*, *C. avellana*, and *Prunus*) were limited. Pastoral activities were significant, as suggested by Cichorieae, fodder plants (*Trifolium* and *Medicago*), and coprophilous fungal spores. By the 6^th^ century BC, wheat and barley cultivation expanded and grazing pressure increased. Wetland areas (including Cyperaceae, *Phragmites australis*, *Sagittaria*, and *Nymphaea alba*) near the *Anaktoron* grew, possibly due to water management [62,63,68].

#### 2.2.3. Sicily

*SS16—Piazza Armerina—Villa Romana del Casale*—The site is a Roman complex that was settled at the end of the 1^st^ century AD, the “*Villa rustica*” (countryside villa), and has continued to be used throughout the centuries to the Late Roman Villa of the 4^th^ century AD, the phase of the luxury house with mosaics. It was included in the UNESCO World Heritage List in 1997. Pollen samples come from five trenches dug in different points across the site and show remarkable floristic diversity, with over 200 taxa identified. Deciduous *Quercus* and *Fraxinus excelsior* lived in mesophilous woods. *O. europaea*, *M. communis*, *Fraxinus ornus,* and other Mediterranean plants grew in warmer sides, and *Prunus* in wetter sides. Several woody plants were probably part of the green of the Villa, such as *Pinus pinea* used for shadow, the exotic *Citrus*, *Platanus*, *Buxus sempervirens*, and also fruit plants like *J. regia*, *C. avellana*, and again *O. europaea* and *Prunus* species. In the *peristilium*, *V. vinifera* was planted for decoration, also allowing it to produce fruits. Cereal fields were spread in the agrarian landscape in the 4^th^ century AD, while Poaceae wild grass group and Cichorieae pollen testify to a well-developed pastoral economy and animal breeding. In the post-Roman period, the spreading of Mediterranean plants together with *Olea*, *Juglans*, and *Castanea*, suggests the development of arboriculture and cultural landscapes in southern Italy [71,73].

*SSI7—Philosophiana (Sofiana)*—During the Roman Imperial period, on the inland route from Catania to Agrigento, Philosophiana was a *statio* a few kilometres from Villa del Casale, to which it was connected by a road. Pollen spectra from samples excavated in a stratigraphic sequence cut in the northern district of the site depict an agro-pastoral system within the Mediterranean landscape. The macchia was present, with *O. europaea* and species of *Pistacia*, *Erica*, *Helianthemum*, and *Cistus*. The mixed oakwood included *C. avellana* and deciduous *Quercus*, while *Pinus* was less common than in the Villa del Casale. Wet environments were limited. Cereal fields were cultivated around the site. The synanthropic plants were common including trampling-resistant plants (e.g., *Plantago*), ruderals, and nitrophilous plants (e.g., *Urtica* and Chenopodiaceae/Amaranthaceae) [73].

*SSI8—Taormina—Teatro greco-romano*—The GreekRoman theatre of Taormina was built in the 3^rd^ century BC during Greek times for theatrical performances, then restructured and enlarged during Roman times to host gladiator games. Pollen analyses were conducted on cores collected within the perimeter of the theatre. The results enhance our understanding of the flora that has been present in and around the area, characterised by species known for their symbolic or ornamental value in classical times. Hedges of *B. sempervirens*, *M. communis*, and *Juniperus* were grown, along with *Rosa* and *Crataegus*, and trees such as *Prunus*, *O. europaea*, *C. sativa*, *J. regia*, *Q. ilex*, and *P. orientalis*. Overall, pollen data indicate the presence of open areas in arid environments, as well as elements from meadows and freshwater habitats. Pollen data also reveal a significantly high presence of *N. alba* in the sediments accumulated in the theatre, suggesting that silt containing its pollen was gathered from wet areas to make the theatre floors [70,71].

*SSI9—Stromboli—San Vincenzo*—Stromboli, a small island in the southern Tyrrhenian Sea, is an active volcano in the Aeolian Archipelago and a UNESCO World Heritage site. Archaeological remains indicate discontinuous occupation in the northeastern sector of the island, likely influenced by environmental changes, with pollen spectra—obtained from stratigraphic profiles and archaeological layers—revealing landscape transformations from the Bronze Age to the present [85]. In the early phase of San Vincenzo-Stromboli, the island was dominated by grasslands with Poaceae and Chenopodiaceae/Amaranthaceae, sparse woodlands and widespread Mediterranean macchia. During the Bronze Age, human occupation developed in a humid phase that supported the growth of Cyperaceae, *P. australis*, *N. alba*, and *Typha latifolia* type. In the Classical Age, the tree cover consisted primarily of deciduous *Quercus* and *P. orientalis*. By the Middle Ages, cereal cultivation and vineyards emerged, and the current wild plant associations, including *Erica* and *Q. ilex*, were established [74].

*SSI32—Morgantina—agorà*—The ancient settlement of Morgantina in central Sicily lasted from the Late Bronze Age until the end of the Roman Republic in the 1st century BC. The ancient *agorà*, the political and commercial centre, was the central place of the city. Pollen samples were taken from a vertical sequence in a trench dug at the centre of this square. Pollen data show an open landscape, with marginal forest cover and shrubby Mediterranean vegetation, scattered water places, cereal fields and synanthropic plants covering margins of fields and houses. The most common trees were deciduous *Quercus*, *F. ornus*, *C. avellana*, and *Salix*, while fruit trees *Prunus*, *O. europaea*, and *J. regia* were also present. The Mediterranean vegetation, with *Erica* and olive trees, was less spread in pre-Roman times, and then increased notably during Roman times. This may be interpreted in different, not antithetic, ways: (a) the use of the site changed, possibly due to a different management of trees and development of the shrubs; (b) olive trees were cared for and cultivated; (c) the climate was milder/warmer thus favouring the development of the Mediterranean vegetation. After a severe decline of Mediterranean vegetation, a relative increase in biodiversity was recorded in post Roman phases.

*SSI39—Mozia—Stagnone di Marsala*—The study concerns the submerged road of Mozia, which connected the Phoenician colony island to the necropolis located on the nearby coast, and also probably led to land devoted to fields and pastures. Pollen was collected about one metre under the water level, from seven short cores near the point where the road joins the island. Pollen shows changes in the floristic/vegetation pattern confirming that in the past the road was sometimes less submerged than today, and sometimes more [86]. These variations suggest significant environmental shifts, likely linked to both natural processes and human activity during the Iron Age and Roman periods. In general, there is prevalence of Mediterranean flora such as Ericaceae, *Juniperus*, *Phillyrea*, *Pistacia*, and *Q. ilex*, evidence of brackish environments as evidenced by Chenopodiaceae/Amaranthaceae pollen, and signs of deforestation. Pollen data attest to the presence of areas subject to human activity for olive cultivation, and, more limited or farther away, for cereal crops. Some differences may reflect the greater proximity to the mainland or the greater input of materials by land in the different cores analysed.

## 3. Discussion

The palynological analysis of archaeological sites is complex and requires cross-disciplinary expertise involving mainly botanists, geologists, and archaeologists. The research is able to provide palaeoecological information, such as pollen flora, vegetation, and environmental inferences, combined with extraordinary details on the anthropogenic influence/impact, land-use, and landscape shaping by anthropogenic activity on a long-time scale. Several factors may complicate such research: dating difficulties and chronological inaccuracy, the scarce content of organic matter, and the poor state of pollen preservation (e.g., [87,88]). Nevertheless, careful sampling of biostratigraphic sequences, pollen extraction treatments involving pollen concentration [89]), and highly detailed analyses (mostly at 1000× magnification) ensure a good success rate in palynological analyses of archaeological sites. Moreover, dealing with interdisciplinary research, pollen data from archaeological sites demonstrate how pollen analysis can serve as *independent* evidence for interpreting different archaeological contexts [90]. The presence of specific pollen indicator taxa, including synanthropic plants (e.g., [31,32,91,92,93]), is not used to confirm what is already known, but rather to demonstrate that pollen data can independently reflect the nature—namely the anthropogenic nature—of the sites (urban, rural, agro-pastoral, etc.) and their uniqueness [20].

The on-site records chosen in this research have returned an excellent floristic picture, which is useful for understanding biodiversity and land-use over the last three millennia. In the following paragraphs, the floristic diversity is analysed, and the potential of assessing anthropogenic pollen in the studied contexts are discussed, supported by statistical analyses highlighting patterns and relationships within the data. Then, a brief comparison between past and present is proposed, aimed at demonstrating the usefulness of palaeoenvironmental research.

### 3.1. Floristic Palaeodiversity of Southern Italy

**Figure 2:**
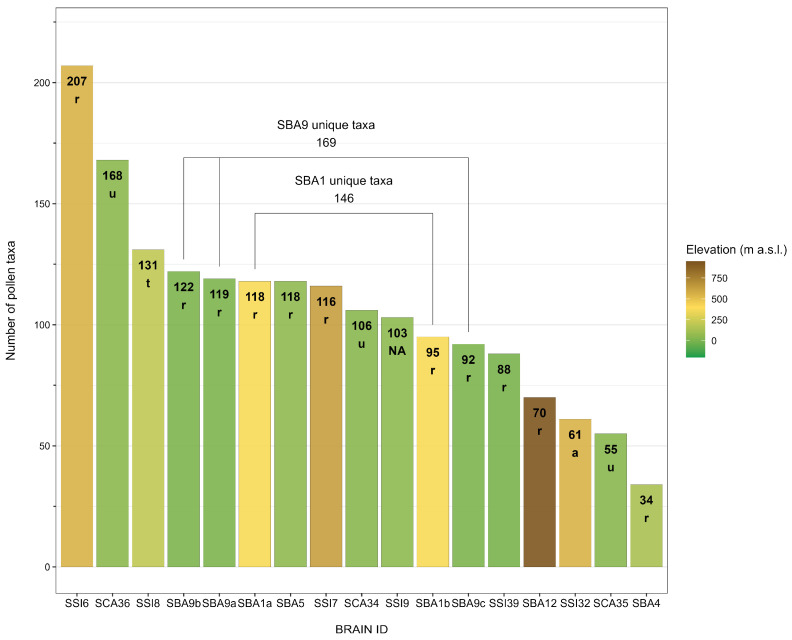
Number of pollen taxa in each examined record. Numbers in the bars indicate the exact number of taxa identified, whereas letters indicate the site’s context as listed in BRAIN: a = *agorà*; r = rural; t = theatre; u = urban; NA = not applicable (multi-phase contexts from the Bronze Age to the present). “Unique taxa” refers to the number of non-duplicate taxa at single sites with multiple records. The bar’s fill colour, from brown to green, marks the elevation gradient. Each site is identified by its BRAIN ID, which consists of three parts: (1) S for Southern; (2) two letters representing the Italian administrative regions (CA = Campania; BA = Basilicata; SI = Sicily); (3) A sequential number automatically assigned when the sites were added to the BRAIN database [60].

The sites had a high floristic diversity as inferred from the significant number of pollen taxa identified (Figure 2)—up to 207 taxa in SSI6—Piazza Armerina—Villa Romana del Casale, with just one site per region having less than 70 taxa.

Every single site examined was either a rural or urban settlement, with the exception of the SSI8 Taormina’s Greek–Roman theatre, which is a monumental building: accordingly, each of them has floristic pollen lists matching significant to high degrees of anthropogenic influence on its native flora.

The results of the group-equalised indicator species analysis [94,95] (Table S2) confirm the palynological information about the floras of the chosen sites.

In Campania, almost all of the indicator pollen taxa highlight the urban and human-induced landscape of the sites: *Platanus orientalis*, an ornamental tree, *Castanea sativa* and *Juglans regia*, fruit trees, *Plantago major* type, a common trampling indicator, and *Cannabis sativa*, a weed or possibly cultivated [31].

In Basilicata, most of the indicator pollen taxa point out both the Mediterranean and agro-pastoral nature of the rural areas studied. *Pistacia lentiscus* L. is a helio-, thermo-, and xerophilous species typical of the evergreen Mediterranean macchia, underlining the spread of Mediterranean vegetation characterising these sites. Papaveraceae pollen (most likely including only pollen of the genus *Papaver*, considering the sites’ context) is an indicator of anthropic pressure [96]. Pollen of the *Trifolium pratense* type [59]—which groups pollen of *T. incarnatum* L., *T. medium* L., *T. ochroleucon* Huds., *T. pratense* L., and *T. subterraneum* L.—and Ranunculaceae (context-wise, likely including *Ranunculus acris* group [97], which comprises pollen of *R. acris* L., *R. bulbosus* L., *R. illyricus* L., *R. lanuginosus* L., *R. monspeliacus* L., *R. muricatus* L., *R. nemorosus* DC., *R. paludosus* Poir., *R. polyanthemos* L., *R. repens* L., *R. sardous* Crantz, and *R. trilobus* Desf.) are indicators of pastoral activities [98]. These plant indicators support the reconstruction of agricultural and human-influenced landscapes made by archaeologists in this region.

In Sicily, the sites are characterised by pollen of the *Thalictrum flavum* type [97], which contains many *Thalictrum* species—namely *T. alpinum* L., *T. aquilegiifolium* L., *T. flavum* L., *T. lucidum* L., *T. minus* L., and *T. simplex* L. However, only *T. minus* is currently present in Sicily [7,99]: it grows at the borders of woods, among coarse rubble or in rocky grasslands, and more generally, in well-lit environments and calcareous soils [100,101]. This marks the environmental context of the sites in which this pollen was found (all of the Sicilian sites except SSI9).

The agro-pastoral nature of the studied sites in Basilicata and Sicily is further testified by pollen taxa that act as indicators for the two combined groups, such as Fabaceae (likely including pollen grains of the genera *Astragalus*, *Lotus*, *Melilotus*, and *Ononis* in this context) and *Avena/Triticum* group, whereas others (e.g., *Cistus*) are more indicative of the strictly Mediterranean environment that characterised them.

### 3.2. Ubiquitous Pollen Taxa in Anthropogenic Environments

In all spectra, pollen taxa linked to anthropogenic environments emerge, including both cultivated species and synanthropic plants, which reflect significant ecological shifts driven by agricultural activities and cultural landscapes [102,103,104].

The cultivation of crops like cereals, olives, and vines, along with the domestication of animals, has led to distinct ecological changes, which are detectable in pollen records. Mercuri and colleagues proposed two indices to identify human presence in archaeological sites: the API and the OJC groups [31,32]. The API (Anthropogenic Pollen Indicators) index includes seven pollen taxa, namely cereals, *Artemisia*, *Centaurea*, Cichorieae, *Plantago*, *Trifolium*, and *Urtica*, which were ubiquitous in Italian archaeological sites. Cereals clearly indicate human presence, while other taxa, such as *Centaurea* and *Plantago*, are typical of disturbed habitats, often linked to grazing practices. The OJC group is based on tree taxa (*Olea*, *Juglans*, and *Castanea*), whose use by humans has promoted their spread. Thus, the index serves as an indicator of arboriculture. Among the indices developed in the Mediterranean basin is the Pollen Disturbance Index (PDI), introduced in Greece by Kouli et al. [105], alongside the approach used by Servera-Vives et al. [96], who introduce anthropogenic pollen indicators for the Balearic Islands, assess potential gradients of human impact on vegetation, and propose region-specific API indicators for Mediterranean islands.

**Figure 3:**
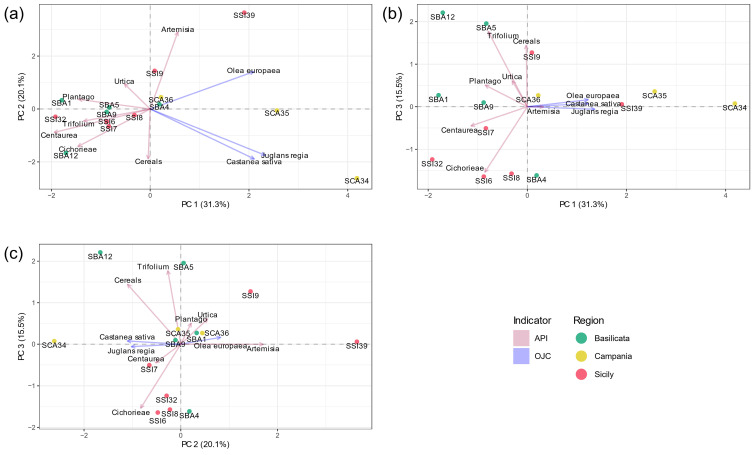
Principal Component Analysis (PCA) showing the distribution of pollen taxa based on the API and OJC groups, indices of anthropogenic environments. Samples from Basilicata (green), Campania (yellow), and Sicily (red) are displayed, with the pollen taxa as variables contributing to the variance. The plots represent the following principal component relationships: (**a**) PC1 vs. PC2, (**b**) PC1 vs. PC3, and (**c**) PC2 vs. PC3. The first principal component (PC) accounts for 31.3% of the total variance, while the second PC captures the second largest variation in the data, explaining 20.1%. The third component, on the other hand, explains 15.5% of the variance.

To better understand the dynamics of anthropogenic pressure on the flora across different temporal and functional contexts, pollen data from sites dating from the Bronze Age to Middle Ages were analysed together. These include a variety of archaeological contexts—such as gardens, farms, and residential areas—each reflecting specific land-use practices which were discussed in the relevant analytical papers (Table S1). Although chronologically and functionally diverse, this comparative approach allows us to explore broader patterns in floristic changes, and human–environment interactions, with a focus on identifying region-specific land-use strategies over time. Pollen data from the anthropogenic pollen of the sites considered for Basilicata, Campania, and Sicily were processed using Principal Component Analysis (PCA) to gather insights on similarities and differences between their contexts in terms of plant composition influenced by human activities (Figure 3).

The components obtained have a distinct ecological–environmental connotation: indeed, the first principal component (PC1) exhibits high loadings for arboreal taxa and low loadings for herbaceous taxa. In the plot of the first two principal components (PC1 vs. PC2; Figure 3a), most of the sites are distributed along the first component, where pollen taxa with high loadings predominantly correspond to sites in Campania, while those with low loadings are mainly associated with other contexts in Basilicata and Sicily. The separation of the Campania sites is clearer in the PC1 vs. PC3 plot (Figure 3b). Statistically, this indicates that the Campania sites are positioned in a way that reflects a specific pattern in the data, primarily driven by the variables associated with cultivated trees. The second principal component (PC2) shows high loadings for some synanthropic herbaceous taxa generally associated with pasture activities (e.g., *Urtica* and *Plantago*), and low loadings for cultivated or potentially cultivated plants (e.g., cereals and *Trifolium*). The third principal component (PC3) is primarily influenced by the same variables as the previous one, but in the opposite direction. Analysing the PC1 vs. PC3 plot (Figure 3b), the samples from Basilicata and Sicily clearly cluster together, with minimal overlap. This suggests that PC1 and PC3 capture significant regional dissimilarities. This separation is clearly delineated by the type of land-use that characterised the regions. Statistically, two Basilicata sites (SBA5 and SBA12) are strongly associated with cereal and legume cultivation, as shown by their distinct positioning in the PC1 vs. PC3 and PC2 vs. PC3 plots (Figure 3b and Figure 3c, respectively). The remaining Basilicata sites and the Sicilian sites, on the other hand, are predominantly characterised by pastureland.

The PCA shows not only regional differences but also captures how these differences are related to temporal and functional variables. For example, the clustering of Campanian sites—Roman urban gardens—reflects a land-use based on arboriculture and ornamental planting. In contrast, the clustering of Basilicata and Sicilian sites, often associated with rural contexts from the Hellenistic and Roman periods, respectively, indicates a more extensive agro-pastoral use of the land. The clustering of sites based on these land-use types reflects significant ecological and cultural differences, highlighting the influence of historical land-use practices on the regional biodiversity.

This methodological framework provides a synthetic way to visualise and compare human activities shaping the landscape across different cultural, chronological, and environmental contexts. The novelty of this approach lies in the integrated statistical treatment of pollen datasets with a high-level of identification, which allows for a comparative interpretation that goes beyond site-specific reconstructions.

### 3.3. Exploring the Link Between Past and Present Flora, and the Role of Morphopalynology

To what extent does today’s landscape retain the imprints of the past? This question invites reflection on how current landscapes—whether urban, rural, or natural—bear traces of historical human activity, cultural changes, and environmental shifts. It encourages a deeper understanding of how past practices, events, and natural processes have shaped the world we live in today [106].

During the Roman period, the use of fruit and ornamental trees reflected cultural choices that led to significant changes in the landscape, as these plants were cultivated not only for their edible fruits but also for aesthetic and symbolic purposes. This is further supported by a notable increase in the presence of *Olea*, *Juglans*, and *Castanea* in the off-site pollen spectra, with particularly high values of *Olea* in archaeological sites of southern Italy [32]. The presence of olive in coastal Mediterranean gardens (SCA34, SCA35, and SCA36) may indicate its origin in the nearby natural Mediterranean forest, olive orchards, or the gardens themselves [107,108,109,110,111]. Debate exists regarding the geographical origins and timing of olive cultivation, as genetic studies and macro-botanical remains often suggest conflicting conclusions. To resolve this ambiguity, it is essential to incorporate additional proxies, such as palynological evidence, which offer valuable insights into the biogeography of key species like the olive [110,112,113,114]. By integrating palynological data with other research approaches, we can better trace the environmental and cultural factors that influenced the geographic expansion of the species.

Pollen data from Stabiae offer valuable insights into how natural ecosystems were altered to suit population needs, with arboriculture emerging as a central aspect of the landscape. In this context, the high variability in walnut pollen size further suggests the possible cultivation of different walnut cultivars [77]. The information derived from palynological studies further highlight how detailed morphological analyses conducted on pollen samples from archaeological sites provide high-quality data that shed light on the plant diversity, including the possibility of identifying the presence of cultivars, hybrids, and distinguish between wild and cultivated varieties [115,116,117,118].

Among the trees valued for their aesthetic qualities, *Platanus orientalis* stands out as a notable example, likely planted for its function as a shade tree. Its broad canopy and striking appearance made it an ideal choice for shaded areas, reflecting its ornamental and practical value. *P. orientalis* was identified in archaeological samples from the Greek–Roman theatre of Taormina [70,71]. This suggests that it was a tree widely used as an ornamental species by the Romans [119] and has been part of the island’s landscape at that time. As such, it is classified as an archaeophyte in Italy, indicating its introduction and cultivation by ancient civilizations [120], and still today, the plane tree is planted in for its resistance to environmental factors, its ability to provide shade, its aesthetic value, long lifespan, and relative ease of maintenance. Today, in Italy, it grows spontaneously in Sicily and southern Italy [99].

Both *P. orientalis* and *Fraxinus ornus* were highly valued by ancient populations, with each species playing a significant role in the environment and economy. During Roman times, *F. ornus* spread. This tree, also known as “manna ash”, has been known since ancient times for its timber, fuel and ornamental purposes. Additionally, the leaves can be used as fodder for livestock in poor-pasture areas. Currently, it is employed for reforestation of poor, arid, calcareous or clay soils, and is thought to have been the special producer of “manna”, the sugary extract from the sap which is extracted by making a cut in the bark.

While much of the previous discussion focused on arboreal species, the morphological identification of herbaceous plants, such as *Nymphaea alba*, also provides valuable insights into a more comprehensive understanding of biodiversity dynamics. The presence of *N. alba* pollen in Hellenistic-Roman period sediments in Sicily is significant evidence, especially considering that this species, which was present on the island in the 3^rd^ century BC, has not been recorded in the region since the 20^th^ century [121]. This evidence highlights the ecological conditions of the island during the Hellenistic-Roman period and provides insight into the natural landscape of the time. It also underscores the complementary value of pollen and floristic analyses in reconstructing biodiversity dynamics. Many wetlands in southern Italy, including Sicily, remain under-investigated [122]. This gap in research further emphasises the potential of pollen analysis to shed light on lesser-explored regions and their ecological histories. In this regard, several factors could have contributed to the disappearance of this species, including human-induced changes and the alteration of freshwater habitats. It is also possible that climatic shifts over the centuries affected the distribution of freshwater species like *N. alba*.

These are examples related to the potential of pollen data in supporting studies that can investigate long-term distributional changes in a species, distinguish between wild and cultivated varieties, as well as understand the arrival of new species in a territory and explore the disappearance of certain habitats and species of interest, thus offering invaluable insights into past environmental dynamics and enhancing our understanding of the current biodiversity.

## 4. Materials and Methods

The research began with a query of the BRAIN database to generate a list of sites in southern Italy where palynological analyses had been conducted. The database allows filtering by region and laboratory where the analyses were performed. From this, the sites from southern Italy with available palynological data were extracted, on which we could guarantee the maximum control (sites studied by the Laboratory of Palynology and Palaeobotany–LPP; Figure 1). Both on-site and off-site records were considered. Following this initial search, specific case studies were chosen for further data elaboration. The data filtered were then organised for a more detailed examination, offering a comprehensive view of the region’s biodiversity across different chronological phases.

A map was created using QGIS version 3.42 [123]. The Corine Land Cover 2018 vector data at a resolution of 100 m was used for the base layer. This data set provides detailed land-cover information for Europe, allowing for a precise representation of land-use types at a regional scale [83]. The sites included in the map were categorised according to the land-cover type they fall within and were assigned the corresponding colour from the legend to match the land-cover classification.

For each site, the presence of distinct pollen series, referring to samples collected for diachronic analysis, was assessed, along with the number of pollen samples analysed within each series. The original pollen counts were used, categorised into “AP = Trees, shrubs and lianas” and “NAP = Herbs”. Pollen analyses of the selected sites were carried out from the 1990s to the present. As a result, many of the pollen spectra did not reflect the most up-to-date botanical nomenclature. Consequently, the pollen lists were revised to incorporate the latest taxonomic updates. In this context, the family Chenopodiaceae/Amaranthaceae was used. Furthermore, Cichorieae refers to fenestrate pollen grains, which belong only to Asteraceae within that tribe [124].

Group-equalised indicator species analysis [94,95] was performed on percentage data for the selected sites, with a priori defined groups based on their geographical region: Basilicata, Campania, Sicily. PCA was performed on percentage data of the selected variables (API and OJC groups—[31,32]), centred and scaled.

Both statistical analyses were carried out with R (v. 4.4.3) [125], and the R package indicspecies [94] (v. 1.8.0) in the case of group-equalised indicator species analysis. Figure 2 and Figure 3 were produced with R and the R packages ggplot2 (v. 3.5.1) [126], ggnewscale (v. 0.5.1) [127], and shadowtext (v. 0.1.4) [128].

## 5. Conclusions

The palynological research in archaeological sites reported in this paper was conducted with the primary objective of contributing to an understanding of the floristic diversity and land management by the populations that occupied each site. The palaeo-floristic lists obtained from pollen analysis have provided an invaluable set of data on the biodiversity of past millennia and on the main habitats and environments found around the sites, which demonstrates how palynology can work at the ‘floristic level’, pointing to improved morphological identifications.

For the time period analysed, the landscape has resulted in being as open as today, with a slight but significant increase during the Roman phases. We suggest that this was probably due to a cultural choice, as there is evidence of an increase in the pollen of plane tree and manna ash, which are known to have been used, among other uses, for decoration. Also, some fruit-bearing trees and cereals indicate a shift towards more organised cultivation of plants, likely reflecting an intensification of agricultural practices. This suggests that during the Roman period, a transformation of the landscape took place, blending aesthetic choices with practical needs to sustain the population. The analysis of indicator pollen taxa across the sites in Campania, Basilicata, and Sicily reveals distinct patterns of human influence on the landscapes, with urban and agricultural indicators in Campania, Mediterranean and pastoral traits in Basilicata, and agro-pastoral characteristics alongside Mediterranean biodiversity in Sicily.

The pollen evidence serves as a reminder of the dynamic nature of ecosystems, where species once abundant in certain areas may disappear over time due to a variety of environmental pressures. Looking ahead, a comparison between the floristic lists obtained in this research and the current flora of the studied areas would provide further valuable insights into the long-term changes in biodiversity and landscape dynamics. Such perspective could enhance our understanding of how human populations influenced local ecosystems over time, shedding light on both cultural practices and environmental changes in the region [75,129], helping to inform the urgent conservation efforts required to protect the Mediterranean’s unique ecosystems for the future [130,131].

This approach paves the way for future research, highlighting the role of palynology not only in reconstructing past environments and human impacts, but also in contributing to landscape archaeology [132,133] and in tackling contemporary and future sustainability challenges [134,135].

## Figures and Tables

**Table 1:** Summary of the main results from pollen analyses in selected sites from Campania, Basilicata, and Sicily: number of pollen records, number of pollen samples per record, pollen count, number of pollen taxa, and arboreal (AP)/non-arboreal (NAP) pollen ratio.

BRAIN ID	Name	No. Pollen Records	No. Pollen Samples	Count	No. Taxa	AP/NAP
SCA34	Stabiae—Villa San Marco	1	9	3114	88	29/71
SCA35	Stabiae—Villa Arianna	1	4	1314	37	30/70
SCA36	Pompeii—Civita Giuliana	1	3	1961	94	9/91
SBA1	Altojanni	2	11–12	4156–5760	118–95	15/85–8/92
SBA4	Difesa S. Biagio	1	23	6901	34	6/94
SBA5	Fattoria Fabrizio	1	12	6532	118	82/18
SBA9	Pantanello (Pizzica Pantanello)	3	16–11–13	6155–3465–3866	129–122–92	9/91–11/89–4/96
SBA12	Torre di Satriano	1	4	1191	70	7/93
SSI6	Piazza Armerina—Villa Romana del Casale	1	45	16,322	207	11/89
SSI7	Philosophiana (Sofiana)	1	12	5397	116	9/91
SSI8	Taormina—Teatro grecoromano	1	9	3640	131	14/86
SSI9	Stromboli—San Vincenzo	1	23	5328	103	13/87
SSI32	Morgantina—agorà	1	10	2883	61	10/90
SSI39	Mozia—Stagnone di Marsala	1	10	819	88	49/51

## Data Availability

The data presented in this study are available on request.

## Supplementary Materials

The following supporting information can be downloaded at: https://www.mdpi.com/article/10.3390/plants14091367/s1, Table S1: List of reference sites in southern Italy with pollen analysis extracted from BRAIN; Table S2: Group-equalised indicator species analysis results.
